# Weak Value Amplification-Based Biochip for Highly Sensitive Detection and Identification of Breast Cancer Exosomes

**DOI:** 10.3390/bios14040198

**Published:** 2024-04-17

**Authors:** Jingru Zhao, Xiaotian Guan, Sihao Zhang, Zhou Sha, Shuqing Sun

**Affiliations:** 1 Institute of Biopharmaceutical and Healthcare Engineering, Shenzhen International Graduate School, Tsinghua University, Shenzhen 518055, China; zjr19@mails.tsinghua.edu.cn (J.Z.);; 2 School of Medicine, Tsinghua University, Beijing 100084, China

**Keywords:** weak value amplification (WVA), biochip, exosomes, breast cancer

## Abstract

Exosomes constitute an emerging biomarker for cancer diagnosis because they carry multiple proteins that reflect the origins of the parent cell. The highly sensitive detection of exosomes is a crucial prerequisite for the diagnosis of cancer. In this study, we report an exosome detection system based on quantum weak value amplification (WVA). The WVA detection system consists of a reflection detection light path and a Zr-ionized biochip. Zr-ionized biochips effectively capture exosomes through the specific interaction between zirconium dioxide and the phosphate groups on the lipid bilayer of exosomes. Aptamer-modified gold nanoparticles (Au NPs) are then used to specifically recognize proteins on exosomes to enhance the detection signal. The sensitivity and resolution of the detection system are 2944.07 nm/RIU and 1.22 × 10^−5^ RIU, respectively. The concentration of exosomes can be directly quantified by the WVA system, ranging from 10^5^–10^7^ particles/mL with the detection limit of 3 × 10^4^ particles/mL. The use of Au NPs-EpCAM for the specific enhancement of breast cancer MDA-MB-231 exosomes is demonstrated. The results indicate that the WVA detection system can be a promising candidate for the detection of exosomes as tumor markers.

## 1. Introduction

Exosomes are lipid bilayer membrane vesicles with a size and diameter ranging from approximately 40–160 nm [1]. Exosomes secreted by different cell lines contain different types of proteins. Notably, exosomes that carry tumor proteins hold promise as novel tumor markers, making them valuable for diagnostic purposes [2]. Exosome-based detection has been explored in various cancers, including ovarian cancer [3,4,5,6], breast cancer [7,8,9,10,11], pancreatic cancer [12,13], glioblastoma [14,15], gastric cancer [16], etc. Among these, breast cancer stands out as a common malignant tumor that significantly impacts women’s health. Its incidence rate continues to rise globally. The epithelial cell adhesion molecule (EpCAM) is highly expressed in nearly all adenocarcinomas and plays a crucial role in the occurrence of the epithelial–mesenchymal transformation of breast cancer cells [8]. Overall, exosomes hold great potential as diagnostic biomarkers, particularly in the context of breast cancer research.

Various detection methods have been explored for exosome analysis, including Colorimetry [17,18], surface plasmon resonance detection [3,19], electrochemical sensing, etc. [11,20]. While numerous exosome detection schemes have been reported, the available capture probes are primarily limited to aptamers and antibodies. These methods are advantageous due to their simplicity and speed, making them suitable for instant clinical diagnosis. However, they also suffer from drawbacks such as low reproducibility and inefficient collection. Using antibodies as the initial exosome collection method may inadvertently lead to the capture of unintended exosome “subpopulations” [21]. Recent studies propose an alternative approach based on the characteristics of exosome phospholipid membranes. Specifically, these membranes can be used to collect exosomes or amplify exosome detection signals. The underlying principle involves the specific interaction of phosphate groups on the exosome lipid bilayer and materials like TiO_2_ [22,23] and Zr-MOF [14,24,25]. The main advantage of this method lies in its ability to efficiently capture exosomes within a short timeframe using the Ti-O-P or Zr-O-P bond [26,27,28]. For instance, TiO_2_ microspheres-based serum exosome isolation achieves a high isolation efficiency of 93.4% [22]. Fe_3_O_4_@TiO_2_ nanoparticles enrich exosomes within 5 min with a capture efficiency of 96.5% [23]. Hydrophilic Fe_3_O_4_@PDA@UIO-66-NH_2_ is employed to consecutively enrich urinary exosomes through the strong bond between Zr and phosphate groups. The recovery of exosomes reached 95% [24]. In summary, utilizing an exosome capture method based on Ti-O-P or Zr-O-P bonds enables the rapid and efficient capture of exosomes.

Metal nanoparticles play an important role in biochemical sensing due to their excellent physical and chemical properties. Among these, gold nanoparticles (Au NPs) find extensive application in biochemical sensing due to their straightforward modification process and excellent photochemical stability. The aptamer, a ligand system developed through in vitro screening and exponential enrichment, exhibits a strong affinity for the target molecule. Consequently, aptamers are increasingly replacing antibodies in biochemical assays. Aptamers can be well modified onto Au NPs through Au-S interaction, and the Au NPs–Aptamer structure plays a pivotal role in specific recognition and signal amplification detection [29,30,31].

The weak value amplification (WVA) was first proposed by Aharonov in 1988 [32] and subsequently verified through optical experiments in 1991 [33]. In 2008, Nature reported the discovery of a laser beam displacement of 0.1 nm using a weak measurement system, which sparked interest in sensing detection based on WVA [34]. Among the various WVA sensing light paths with different structures, the reflective light path stands out. It is capable of detecting interface-based immune reactions, etc. [35]. Notably, this sensing system is built on a glass substrate and does not require gold plating, significantly reducing the inspection costs compared to surface plasmon resonance (SPR). To the best of our knowledge, the combination of the WVA system with Zr-ionized biochips has not been reported.

In this work, we develop a glass biochip with a reflective optical detection system based on the WVA for the highly sensitive detection and identification of breast cancer exosomes. The sensitivity and stability of the WVA system are evaluated by utilizing a concentration gradient of sodium chloride solution. Then, the Zr-ionized glass substrate can specifically bind to the phosphate group on the exosome phospholipid bilayer. Notably, both aptamer and antibody capture probes require a certain incubation time for exosome capture, ranging from 40 min to 1.5 h. In contrast, our biochip only requires a 10 min incubation time, significantly reducing the experimental duration. Moreover, EpCAM aptamer-modified Au NPs (Au NPs-EpCAM) can specifically recognize proteins on breast cancer MDA-MB-231 exosomes through the affinity between the aptamer and protein, thus achieving effective tumor screening. In summary, our developed WVA biosensing strategy enables the accurate detection of EpCAM-positive exosomes with high sensitivity and specificity. This approach holds excellent potential for detecting exosomes as tumor markers.

## 2. Materials and Methods

### 2.1. Reagents

Sulfuric acid (H_2_SO_4_) was obtained from Xilong Scientific, Guangzhou, China. Hydrogen peroxide (H_2_O_2_) was purchased from Shanghai Lingfeng Chemical Reagent Co., Ltd., Shanghai, China. Ammonia solution (NH_3_·H_2_O), zirconyl chloride octahydrate (ZrOCl_2_), and Tris (2-carboxyethyl) phosphine (TCEP) were purchased from Shanghai Aladdin Biochemical Technology Co., Ltd., Shanghai, China. Sodium chloride (NaCl) and chloroauric acid (HAuCl_4_) were obtained from Shanghai Mackin Biochemical Co., Ltd., Shanghai, China. Sodium dodecyl sulfate (SDS) was purchased from Damao Chemical Reagent Factory, Tianjin, China. Hydroxylammonium chloride (HONH_3_Cl) was obtained from Sinopharm Chemical Reagent Co., Ltd., Shanghai, China. Sodium citrate (SC) was obtained from Fuchen Chemical Reagent Co., Ltd. Tianjin, China. Polydimethylsiloxane (PDMS) was purchased from The Dow Chemical Company, Midland, MI, USA. Aptamer was purchased from Sangon Biotech (Shanghai) Co., Ltd., Shanghai, China. Prostate specific antigen (PSA) and alpha fetoprotein (AFP) were purchased from Shanghai Linc-Bio Science Co., Ltd., Shanghai, China. Triple negative breast cancer cells (MDA-MB-231) were obtained from the China Center for Type Culture Collection. Exosome-depleted fetal bovine serum and RPMI-1640 medium were purchased from Gibco Co., Ltd. (Waltham, MA, USA).

CD63: 5′-SH C6-CACCCCACCTCGCTCCCGTGACACTAATGCTA-3′

Scrambled CD63 (SCD63): 5′-SH C6-ACTCTTCACCCGCACATTCGCATCGCCCAAGC-3′

EpCAM: 5′-SH C6-CACTACAGAGGTTGCGTCTGTCCCACGTTGTCATGGGGGGTTGGCCTG-3′

### 2.2. Instrumentation

Transmission electron microscopy (TEM) images were obtained using the FEI spirit T12. Thermal field emission scanning electron microscope (SEM) images were recorded using an Apreo 2S. X-ray photoelectron spectroscopy (XPS) spectra were acquired on a PHI 5000 VersaProbe Ⅱ. Ultraviolet–visible absorption (UV-Vis) spectra were acquired on the Shimadzu, Japan, UV-2450. Dynamic light scattering (DLS) data were measured by using the Malvern, Zetasizers Nano ZS. Nanoparticle Tracking Analysis (NTA) data were measured by using a Malvern NanoSight NS300.

### 2.3. Preparation of Au NPs and Au NPs–Aptamer

The preparation process of Au NPs has been reported [36]. We adopted the seed growth method to prepare Au NPs. The gold seeds were obtained by reducing HAuCl_4_ (29.43 mM, 1 mL) with SC (14.55 mM, 10 mL) at 110 °C in ultrapure water (99 mL) for 20 min. Based on gold seeds (1.2 mL), Au NPs can be obtained by reducing HAuCl_4_ (3.52 mM, 1 mL) with HONH_3_Cl (40 mM, 1 mL) in ultrapure water (20 mL). Eventually, we dispersed the Au NPs in the SDS (0.3%, 5 mL) solution for future use.

The preparation process of Au NPs–Aptamer is as follows: add TCEP (12 mM) to a tube of sulfhydryl DNA (1 OD) and incubate it for 1 h. The prepared Au NPs (9 mL) are centrifuged at 5000 rpm (8 min) twice, and dispersed in ultrapure water after removing the supernatant. The Au NPs are then mixed with the aptamer and incubated overnight at room temperature using a tilt rotator. The same volume of NaCl (0.3 M) is added to the Au NPs–Aptamer solution in 10 fractions, with an interval of 30 min each time. The final concentration of the NaCl in the Au NPs–Aptamer solution is 0.15 M. Au NPs–Aptamer is centrifuged and finally dispersed in ultrapure water and stored at 4 °C for later use.

### 2.4. Preparation of MDA-MB-231 Exosomes

The supernatant of the cell culture was ultracentrifuged three times, 300× *g* (10 min), 2500× *g* (20 min), and 10,000× *g* (30 min), respectively. After filtering (0.22 μm) the centrifuged supernatant, further centrifugation at 4 °C, 120,000× *g* (90 min) was performed. The supernatant was removed, and the residue was dispersed in PBS solution.

### 2.5. Preparation of PDMS Patches

A PDMS microfluidic patch was made via soft lithography. The process is briefly described as follows: firstly, the layout was drawn by using software, and etched on a chrome-plated and photoresist-coated quartz glass substrate to make a mask. The pattern on the mask was then transferred to a clean silicon wafer using photolithography to form a mold. Subsequently, the liquid PDMS and curing agent were fully mixed in a ratio of 10:1 and poured on the silicon wafer mold. After the bubbles had discharged, it was placed in an oven at 70 °C for 2 h. The PDMS patch was finally peeled off from the silicon wafer. After slicing and punching, it was combined with the injection and discharge pipes to form a PDMS microfluidic patch. The size of the runner was 15 mm × 3 mm × 100 μm.

### 2.6. Preparation of Glass-Based Biochips

K9 prisms (20 mm × 20 mm × 20 mm) were bought from Heng Yang Guang Xue. The prism was soaked in a piranha lotion solution with a volume ratio of concentrated sulfuric acid to hydrogen peroxide of 7:3 (this solution is highly sensitive to organic matter and may boil, so it needs to be handled with care) and cleaned with ultrapure water. Then, it was placed in RCA-1 solution (NH_3_·H_2_O:H_2_O_2_:H_2_O = 1:1:5) at 80 °C, followed by rinsing using a large amount of ultrapure water. The cleaned prism was then infiltrated into 5 mM zirconia solution and kept in the oven at 70 °C for 2 days. After removal, it was ultrasound cleaned in ultrapure water for 1 min, then washed with a large amount of ultrapure water and dried with nitrogen. The construction of the entire chip system was completed by binding the glass-based biochip and the microfluidic patch prepared above.

### 2.7. The WVA Detection System Construction and Sensing Process

**Figure 1:**
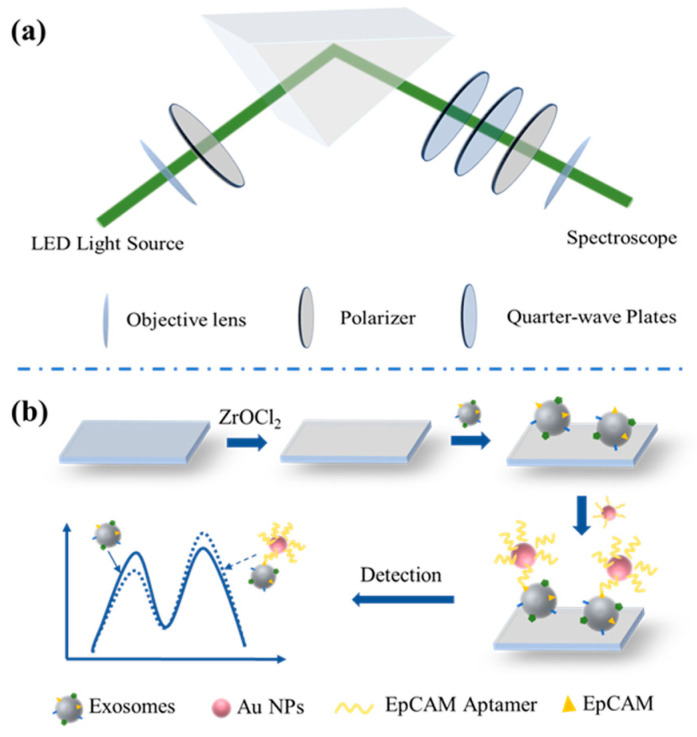
The schematic diagram for (**a**) the WVA detection system and (**b**) the sensing principle of the biochip.

A schematic diagram of the detection system based on WVA is shown in Figure 1a. A 530 nm green LED light source (Thorlabs, Newton, NJ, USA, M530F2) passed through a lens, a pre-selective polarizer, and a K9 prism to form a total reflection on the K9 prism, and then the light source passed through two quarter-wave plates (Thorlabs, WPQ10ME-532) and a post-selective polarizer before finally reaching the spectrometer (Ocean Optics, OCEAN-FX-VIS-NIR-ES, 350–1000 nm wavelength range). The fiber used in the optical path was from Ocean Optics, QP400-1-VIS-NIR. A schematic diagram of the sensing detection process is shown in Figure 1b. First, the cleaned K9 prism was Zr-ionized. Due to the coordination of Zr-O-P, the interface captured exosomes and gave different double peak pointer (DPP) signals. Au NPs–Aptamer was pumped into the channel and due to the specific binding of aptamers to proteins on exosomes, the DPP signal was altered.

## 3. Results and Discussion

### 3.1. The Principle of the WVA Detection System

The DPP signal obtained by the WVA detection system was phase-correlated [35]. The following is the inference of the detection optical path constructed based on the WVA: The expression of the pre-selection state is as follows:

(1) $$|\psi_{i}\rangle =sin\alpha |H\rangle +cos\alpha |V\rangle$$

where H is horizontally polarized light; V is vertically polarized light; and α is the pointing angle. The expression of the post-selection state is as follows:

(2) $$|\psi_{f}\rangle =\cos\left(\alpha +\beta \right)|H\rangle +\sin\left(\alpha +\beta \right)e^{i\varphi }|V\rangle$$

where β is the orthogonal direction angle between the post-selected and the pre-selected polarization state. *φ* is the phase difference formed in the optical path. The polarization operator of the observable A is as follows:

(3) A=|H〉 〈H|−|V〉 〈V|

The expression of the weak value of the observable A_W_ is as follows:

(4) $$A_{w}=\frac{\langle \psi_{f}|\hat{A}|\psi_{i}\rangle }{\langle \psi_{f}|\psi_{i}\rangle }=\frac{sin\alpha \cos\left(\alpha +\beta \right)+cos\alpha \sin\left(\alpha +\beta \right)e^{i\varphi }}{sin\alpha \cos\left(\alpha +\beta \right)-cos\alpha \sin\left(\alpha +\beta \right)e^{i\varphi }}$$

Substituting $\gamma =cot\alpha \tan\left(\alpha +\beta \right)$ into the weak value expression, the weak value imaginary part expression can be obtained as follows:

(5) $${I_{m}A}_{w}=\frac{1+\gamma e^{i\varphi }}{1-\gamma e^{i\varphi }}\approx \frac{2\gamma sin\varphi }{1+\gamma^{2}-2\gamma cos\varphi }$$

After substituting the expression of the momentum and the imaginary part of the weak value, the expression between the shift of the center wavelength (CW) and phase change can be obtained as follows:

(6) $$\delta \lambda =\frac{{-2\pi k(\Delta \lambda )}^{2}}{\lambda_{0}}{I_{m}A}_{w}=\frac{{-4\pi k(\Delta \lambda )}^{2}\gamma sin\varphi }{\lambda_{0}(1+\gamma^{2}-2\gamma cos\varphi )}$$

Through the above derivation, it can be found that the change of phase affects the change of the CW, and the change of the interface affects the change of phase. Therefore, a shift in the CW implies a change in the interface.

### 3.2. Evaluation of the Sensitivity and Stability of the WVA Detection System

**Figure 2:**
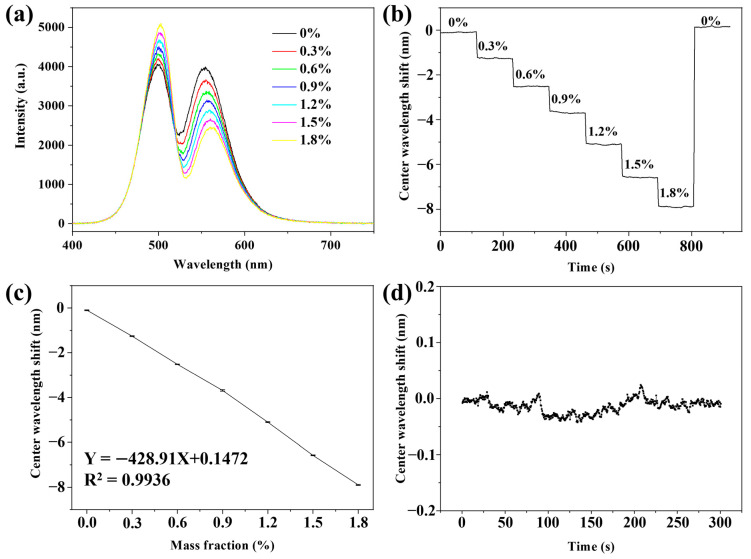
Evaluation of the sensitivity and stability of the WVA detection system. (**a**) DPP graph, (**b**) Kinetic curve, and (**c**) Linear fitting curve of NaCl solution with different mass fractions; (**d**) Graph of system stability test after continuously passing in the ultrapure water for 300 s.

To establish an assay protocol for quantitative exosome analyses, we first evaluated the sensitivity and stability of the WVA system. We employed different concentrations of NaCl solutions, ranging from 0% to 1.8%. By utilizing the relation formula between concentration and refractive index, we determined that the change in refractive index (Δn) within this concentration range was 0.002646. The DPP graph of NaCl solution with different concentrations is depicted in Figure 2a, where DPP changes are observed with concentration variations. We converted the DPP signal into a CW digital signal through our program, resulting in the kinetic curve of the CW shift over time for different NaCl solution concentrations, as shown in Figure 2b. The solution flow rate was at 50 μL/min for a duration of 2 min. From Figure 2b, within this 2 min period, there was a minimal shift in the CW of the solution. However, a gradient change was evident between solutions of different concentrations. We performed a linear fit to the kinetic curve, and the results are presented in Figure 2c. Specifically, the various NaCl solution concentrations (ranging from 0–1.8%) exhibited shifts of 1.16, 2.41, 3.58, 5.00, 6.48, and 7.79 nm, respectively. The high R^2^ value of 0.9936 indicates a robust linear relationship between the concentration of NaCl solution and the shift in CW. Through the sensitivity calculation formula,

(7) $$S_{n}=\Delta \lambda /\Delta n$$

the sensitivity (S_n_) of the WVA detection system was 2944.07 nm/RIU. Following the sensitivity testing of the system, the next step involved assessing its stability. We achieved this by continuously injecting ultrapure water into the flow channel for a duration of 300 s. During this process, we recorded a CW data point every half second and plotted the results, as depicted in Figure 2d. To quantify stability, we calculated the standard deviation (σ) of CW data points, which was determined to be 0.012 using the following formula,

(8) $$R_{n}=3\sigma /S_{n}$$

The resolution (R_n_) of the WVA detection system was 1.22 × 10^−5^ RIU.

### 3.3. Characterization of Au NPs, Au NPs–Aptamer, and Exosomes

**Figure 3:**
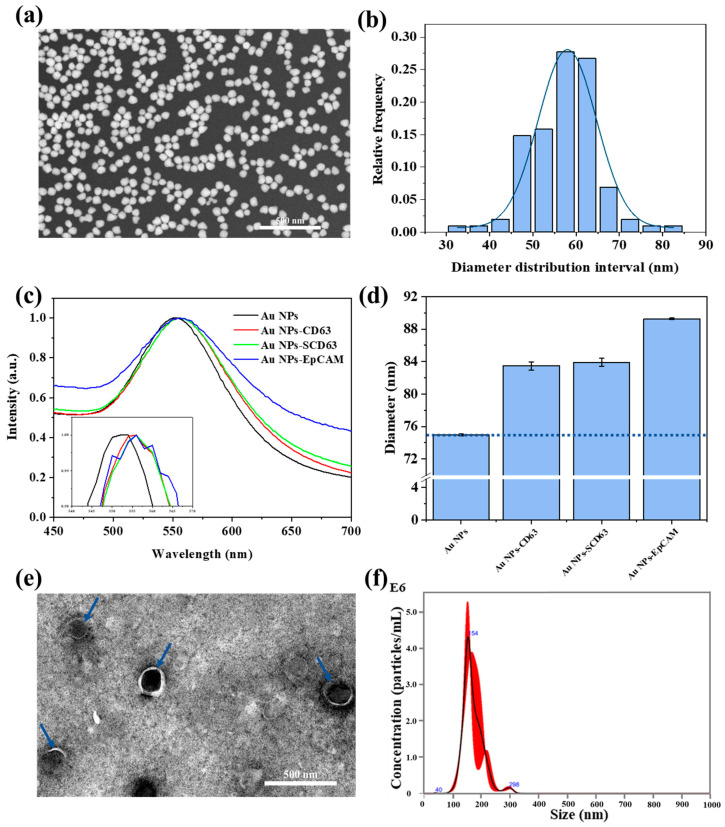
Characterization of Au NPs, Au NPs–Aptamer, and exosomes. (**a**) SEM image, and (**b**) Size distribution histogram of Au NPs; (**c**) UV-Vis spectra of Au NPs and Au NPs–Aptamer; the inset is a magnified view of the portion of the strongest absorption peak; (**d**) Hydrated particle size histogram of Au NPs and Au NPs–Aptamer; (**e**) TEM image, and (**f**) Size distribution via NTA analysis of MDA-MB-231 exosomes.

Au NPs–Aptamer interactions and their impact on the morphology and structure of Au NPs were investigated. A SEM image (Figure 3a) reveals that Au NPs exhibit a homogeneous and spherical shape; the size distribution diagram, obtained by analyzing the size of Au NPs, is shown in Figure 3b. As shown in Figure 3b, most of the size distribution of Au NPs falls within 45–65 nm, with an average size of approximately 58 nm. The curve in Figure 3b represents a Gaussian fitting curve of the Au NPs size distribution histogram. Then, we characterized the Au NPs and Au NPs–Aptamer using UV-visible spectroscopy. The results are shown in Figure 3c. Through normalization processing, the UV-visible spectrum absorption peak of Au NPs was 552 nm, and the absorption peaks of Au NPs-CD63, Au NPs-SCD63, and Au NPs-EpCAM were all 556 nm. Compared with the Au NPs, the Au NPs–Aptamer absorption spectra had a redshift of about 4 nm, indicating that the aptamer was successfully modified on the Au NPs. The same peak shift was observed in the UV-vis spectra of Au NPs modified using different aptamers, despite the use of three different aptamers. One possible reason for this similarity could be the limited resolution of the UV-visible spectrometer. Additionally, in Figure 3c’s inset, we observe that the UV-vis spectra of Au NPs-CD63 and Au NPs-SCD63 are identical, while Au NPs-EpCAM exhibits a broader peak width. This broader peak width for Au-EpCAM compared to Au-CD63 and Au-SCD63 highlights the differences between the EpCAM aptamer and the other two aptamers. We next analyzed the hydrated particle sizes of Au NPs, Au NPs-CD63, Au NPs-SCD63, and Au NPs-EpCAM. As shown by the blue dotted line in Figure 3d, the hydrated particle size of Au NPs-CD63, Au NPs-SCD63, and Au NPs-EpCAM was larger than that of Au NPs, indicating that the aptamer was successfully modified on Au NPs. Among the three different aptamers, the EpCAM aptamer exhibited the longest chain length, while the chain lengths of CD63 and SCD63 were similar. Regarding hydrated diameter, the Au NPs-EpCAM was the largest, whereas the Au NPs-CD63 and Au NPs-SCD63 showed nearly identical sizes. The TEM image of exosomes is shown in Figure 3e; it shows that the average size of exosomes was 150 nm as indicated by the blue arrows in the image. The exosome counting was carried out via NTA, and as shown in Figure 3f, the concentration was approximately 1.53 × 10^9^ particles/mL, and the size distribution was 40–298 nm. The size of most exosomes was 154 nm, which is consistent with the size distribution of exosomes in the literature.

### 3.4. Zr-Ionized Biochip and Au NPs–Aptamer 

**Figure 4:**
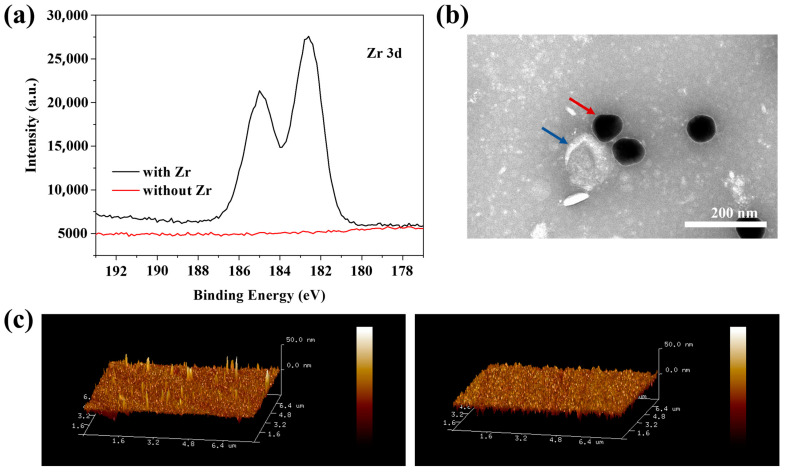
Characterization of glass-based Zr ionization, Au NPs–Aptamer identifying, and Zr-ionized substrate trapping exosomes. (**a**) Zr 3d XPS spectra of the glass substrate with or without Zr ionization; (**b**) TEM image of Au NPs–Aptamer bounded exosomes; (**c**) (7.8 μm × 7.8 μm) 3D AFM image of blank (**right**) and Zr-ionized glass substrate with captured exosomes (**left**).

We investigated the feasibility of Zr-ionized substrates for capturing exosomes and Au NPs–Aptamer for identifying exosomes. After obtaining Au NPs, Au NPs–Aptamers, and exosomes, we prepared the Zr-ionized biochips. In simple terms, a Zr-ionized biochip was created by soaking glass substrates in ZrOCl_2_. We further characterized it using XPS. As shown by the black line in Figure 4a, after Zr ionization on the glass substrate, Zr 3d peaks appeared at 182.7 eV and 185 eV, respectively, illustrating that the Zr ionization modification on the glass substrate was successful. The Zr-ionized glass substrate was characterized by XPS again after the cleaning step. The red line in Figure 4a shows the disappearance of the Zr 3d peak, indicating that the glass substrate can be reused after the cleaning step. CD63 is a common protein on most types of exosomes, so the feasibility of this identification method can be verified via Au NPs modifying with CD63 aptamers. Figure 4b shows the TEM image of the specific identification between Au NPs-CD63 and exosomes; the red arrows and the blue arrows refer to Au NPs and exosomes, respectively. Figure 4b indicates the specific binding of the CD63 aptamer with CD63 on the exosomes and further illustrates the feasibility of the Au NPs–Aptamer identifying exosomes. AFM is one of the most important tools for the characterization of exosomes, and we used it to evaluate the feasibility of utilizing biochips to capture exosomes. Figure 4c shows the AFM images of a blank Zr-ionized substrate (right) and a Zr-ionized substrate with captured exosomes (left); the white protrusions are exosomes. Comparing AFM images proved that the biochips effectively captured exosomes.

### 3.5. Gradient Detection of Exosomes by Using the WVA Detection System

**Figure 5:**
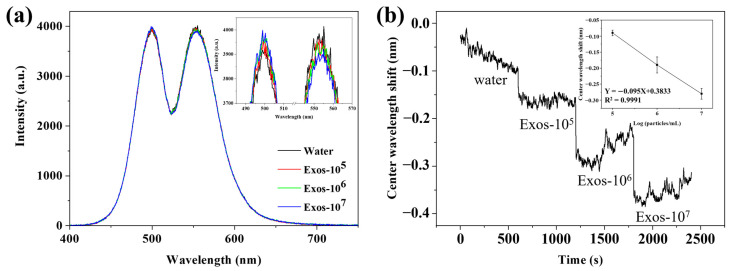
Gradient detection of exosomes by using WVA system. (**a**) DPP signal detected by WVA system sensing and the inset is an enlarged picture of part of the DPP signal; (**b**) Kinetic curve of exosome sensing detection with different concentrations, and the inset is the correlation analysis of CW shift and logarithm of exosome concentrations.

To demonstrate the feasibility of the WVA system, our initial step involved detecting exosomes with varying concentration gradients. Figure 5a shows that different DPP signals were obtained from different concentrations of exosomes. The inset is an enlarged picture of Figure 5a; there is a corresponding shift in the DPP signal of different concentrations of exosomes, indicating that the WVA system can capture the signal response of 10^5^–10^7^ particles/mL exosomes. The DPP signal was converted into a CW shift signal, and the results are shown in Figure 5b; it is the kinetic curve of the exosome sensing picture, and changes in the CW shift between different concentrations of exosomes over time can be seen. The inset is the correlation analysis of CW shift and logarithm of exosome concentrations. Following the concentration changes, the CW shifts 0.09 nm, 0.19 nm, and 0.28 nm respectively. A linear relationship can be established between the CW shift and the logarithm of exosome concentrations ranging from 10^5^–10^7^ particles/mL, with a linear equation of Y = −0.095X + 0.3833. The correlation coefficient R^2^ was 0.9991, indicating that the linear relationship was good. It can be estimated that 3 × 10^4^ particles/mL was the lowest detected concentration.

### 3.6. Evaluation of the Specific Capture and Identification of Exosomes by Using the WVA Detection System

**Figure 6:**
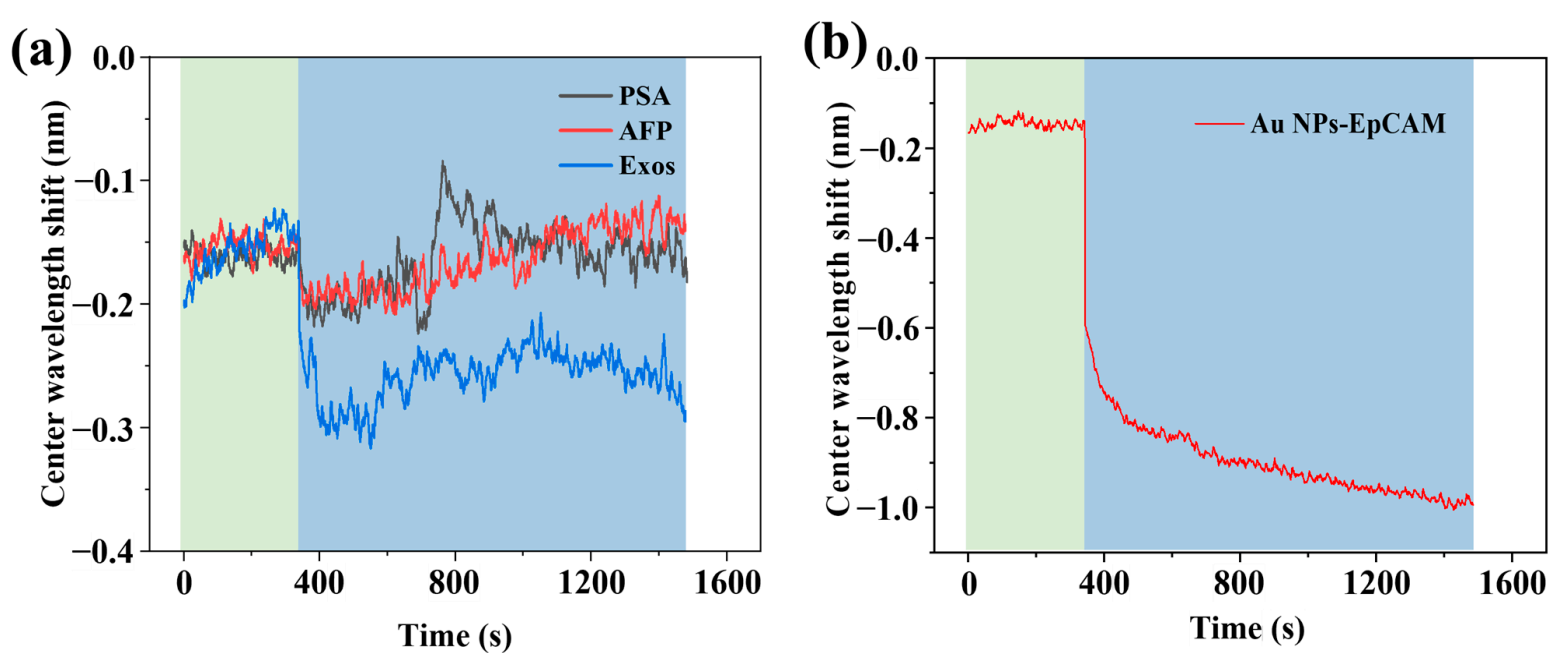
Evaluation of the specific capture and identification of exosomes by using the WVA detection system. (**a**) Kinetic curve of anti-interference performance for Zr-ionized biochip; (**b**) Kinetic curve of specific recognition of EpCAM on MDA-MB-231 exosomes.

Under the condition of detecting exosomes with concentration gradients, the designed WVA system was used for the identification of exosomes with Au NPs–Aptamer, and the results are shown in Figure 6a. PSA, AFP, and exosomes were passed in the flow channel, while the green and blue areas indicate the water and sample, respectively. The CW of the PSA (5 ng/mL) and AFP (25 ng/mL) solutions were shifted by 0.03 nm and 0.02 nm compared to the ultrapure water; their shifts were less than three standard deviations. Exosomes (10^5^ particles/mL) had a shift of 0.1 nm, i.e., more than three standard deviations. We conducted tests by passing PSA and AFP into the biochip, demonstrating that our designed biochip efficiently captures exosomes while excluding interference from common proteins. Importantly, we aimed to confirm that the CW shift is due to specific recognition rather than nonspecific adsorption. To address this, we present results in Figure S1, involving Au NPs modified with CD63 and SCD63 aptamers. Finally, we detected the breast cancer tumor target EpCAM on MDA-MB-231 exosomes, and the results are shown in Figure 6b. The green and blue regions represent ultrapure water and the sample, respectively. When passing the Au NPs-EpCAM solution, the signal shifted by about 0.85 nm, which suggests that this assay can effectively detect EpCAM on MDA-MB-231 exosomes.

## 4. Conclusions

In summary, an optical WVA biosensor has been developed for the highly sensitive detection and specific recognition of EpCAM-positive exosomes based on Zr-ionized biochip and Au NPs–Aptamer. The sensitivity and resolution of this WVA system are 2944.07 nm/RIU and 1.22 × 10^−5^ RIU, respectively, which are sufficient for the detection of exosomes. 

Typically, although numerous exosome detection schemes have been reported, the available capture probes are primarily limited to aptamers and antibodies. Several different exosome detection methods are presented in Table S1, and three indicators are compared: capture probes, exosome incubation time, and detection limits. Our capture approach differs significantly from previous methods; it relies on Zr-ionized substrates. Notably, both aptamers and antibodies require a certain incubation time for exosome capture, ranging from 40 min to 1.5 h, sometimes even requiring specific incubation temperatures. In contrast, our substrate only needs a 10 min incubation time, significantly reducing the experiment’s duration. The biochip can effectively capture exosomes through the Zr-O-P bond showing a good linear relationship in the detection range of 10^5^–10^7^ particles/mL, and the detection limit of exosomes is 3 × 10^4^ particles/mL. Even without signal amplification methods, our current detection limit surpasses that of most existing detection techniques. Overall, we have developed an efficient and highly sensitive method for detecting exosomes. 

Finally, we applied the WVA system for breast cancer exosome detection. Au NPs-EpCAM can specifically recognize EpCAM on MDA-MB-231 exosomes. In the next study, we hope to manufacture a commercial test prototype of the WVA system and use it in the clinic.

## Data Availability

The data supporting the findings of this study are available within the paper.

## Supplementary Materials

The following supporting information [11,37,38,39,40,41,42,43] can be downloaded at: https://www.mdpi.com/article/10.3390/bios14040198/s1, Figure S1: Sensing detection for specific recognition of exosomes; Table S1: Comparison of the analytical performances with other reported methods.
