# Supplementary material for: Weak Value Amplification-Based Biochip for Highly Sensitive Detection and Identification of Breast Cancer Exosomes
Source: Biosensors (Basel). 2024 Apr 17;14(4):198. doi: 10.3390/bios14040198 (PMC11048322; doi:10.3390/bios14040198)
Supplement: Supplementary file 1 [file biosensors-14-00198-s001.zip › biosensors-2950872-supplementary.pdf]

biosensors

MDPI

Article

Weak Value Amplification Based Biochip for Highly Sensitive Detection and Identification of Breast Cancer Exosomes

Jingru Zhao [1,2], Xiaotian Guan [1], Sihao Zhang [1], Zhou Sha [1] and Shuqing Sun [1,*]

[1] Institute of Biopharmaceutical and Healthcare Engineering, Shenzhen International Graduate School, Tsinghua University, Shenzhen 518055, China; zjr19@mails.tsinghua.edu.cn (J.Z.); gxtcqmu@163.com (X.G.); 18110028527@163.com (S.Z.); shaz21@mails.tsinghua.edu.cn (Z.S.)

[2] School of Medicine, Tsinghua University, Beijing 100084, China

* Correspondence: sun.shuqing@sz.tsinghua.edu.cn

1. Au NPs-CD63 specific recognition CD63 on exosomes

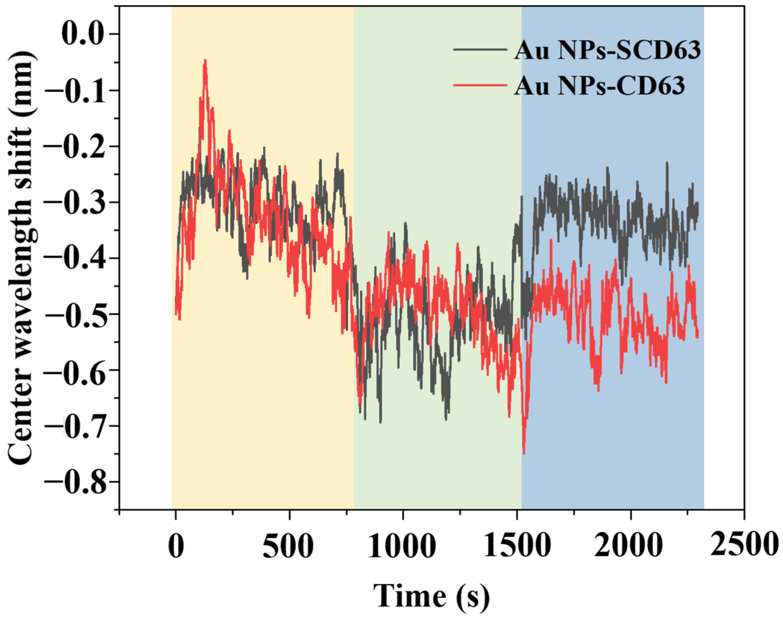

Figure S1. Sensing detection for specific recognition of exosomes

Importantly, we aimed to confirm that the CW shift was due to specific recognition rather than nonspecific adsorption. To address this, we present results in Figure S1, involving Au NPs modified with CD63 and SCD63 (scrambled CD63) aptamer. CD63 is a common protein found on exosomes. The Au NPs modified with CD63 effectively recognized exosomes, while those modified with SCD63 failed to do so. In Figure S1, the black curve represents the results for Au NPs-SCD63, and the red curve corresponds to Au NPs-CD63. The yellow region shows the curve passing with ultrapure water, the green region represents curves introducing Au NPs-CD63 or Au NPs-SCD63, and both curves exhibit nearly identical CW displacement. The blue region represents further flushing with ultrapure water to remove excess unbound Au NPs-CD63 and Au NPs-SCD63 solutions. Notably, the Au NPs-SCD63 curve almost returns to the initial CW shift observed upon introducing ultrapure water, indicating that Au NPs-SCD63 did not bind to exosomes and did not exhibit strong nonspecific adsorption. Conversely, the red curve demonstrates that the final ultrapure water curve closely matches the Au NPs-CD63 curve, and the overall shift of about 0.2 nm occurred, confirming effective exosome recognition. These results

highlight that in Au NPs modified with positive aptamer, exosomes can be efficiently recognized, while negative control aptamer does not cause significant nonspecific adsorption-induced CW shifts.

2. Comparison of the analytical performances

Table S1. Comparison of the analytical performances with other reported methods.

No	Method	Capture Probe	Exosome Incubating Time	LoD (Exosomes/mL)
[11]	Electrochemical	Aptamer	1 h	3.4×10^{3}
[37]	Electrochemical	Antibody	45 min	2×10^{5}
[38]	Fluorescence	Aptamer	NA	1×10^{8}
[39]	Fluorescence	Aptamer	1.5 h	7.19×10^{6}
[40]	Fluorescence	Aptamer	30 min	1.6×10^{5}
[41]	SPR	DNA	1 h	5×10^{3}
[42]	SPR	Aptamer	40 min	5.6×10^{5}
[43]	SERS	Antibody	1.5 h	1.6×10^{5}
This work	WVA	Zr-ionized	10 min	3×10^{4}
